# Development and psychometric testing of a questionnaire for the Korea Youth risk behavior survey to assess physical activity behaviors

**DOI:** 10.1186/s12889-024-19216-z

**Published:** 2024-06-24

**Authors:** Bomi Park, Hye Ah Lee, Yoonhee Shin, Yeonjae Kim, Hyunjin Park, Seunghee Jun, Ui Jeong Kim, Kyungwon Oh, Sunhye Choi, Yangha Kim, Hyesook Park

**Affiliations:** 1 Department of Preventive Medicine, College of Medicine, Chung-Ang University, Seoul, Korea; 2 Clinical Trial Center, Ewha Womans University Mokdong Hospital, Seoul, Korea; 3 Advanced Biomedical Research Institute, Ewha Womans University Seoul Hospital and College of Nursing, Ewha Womans University, Seoul, Korea; 4 College of Nursing, Ewha Womans University, Seoul, Korea; 5 Department of Preventive Medicine, College of Medicine, Ewha Womans University, Seoul, Korea; 6 Graduate Program in System Health Science and Engineering, Ewha Womans University, Seoul, Korea; 7 Division of Health and Nutrition Survey and Analysis, Bureau of Chronic Disease Prevention and Control, Korea Disease Control and Prevention Agency, Cheongju, Korea

**Keywords:** Adolescent, Exercise, Survey, Questionnaire, Reliability, Validity

## Abstract

**Background:**

Physical activity is essential for physical, mental, and cognitive health. Providing evidence to develop better public health policies to encourage increased physical activity is crucial. Therefore, we developed an in-depth survey as part of the Korea Youth Risk Behavior Survey to assess the current status and determinants of physical activity among Korean adolescents.

**Methods:**

We developed an initial version of the questionnaire based on a review of validated questionnaires, recent trends and emerging issues related to adolescent physical activity, and the national public health agenda pertaining to health promotion. Content validity was confirmed by a panel of 10 experts. Face validity was confirmed through focus group interviews with 12 first-year middle school students. The test-retest reliability of the questionnaire was evaluated by administering it twice, approximately two weeks apart, to a sample of 360 middle and high school students. Additionally, the frequency or average number of responses was analyzed in a sample of 600 students who participated in the initial test-retest reliability evaluation of the questionnaire developed in this study.

**Results:**

Through item pool generation and content and face validity test, the final 15 questionnaire items were developed across five themes: levels of physical activity, school sports club activities, transportation-related physical activity, physical activity-promoting environments, and factors mediating physical activity. The test-retest reliability ranged from fair to substantial. Results from the newly developed survey reveal that only a minority of adolescents engage in sufficient physical activity, with only 17.2% and 21.5% participating in vigorous and moderate-intensity activities, respectively, for at least five days per week. Among school-based activities, 44.3% of students do not participate in school sports clubs due to reasons including absence of clubs and disinterest in exercise. The major motivators for physical activity are personal enjoyment and health benefits, whereas preferences for other leisure activities and academic pressures are the predominant barriers.

**Conclusions:**

This study developed valid and reliable in-depth survey items to assess physical activity among Korean youths. It will hopefully enhance our understanding of adolescent physical activity, offering essential preliminary evidence to inform the development of public health strategies aimed at promoting adolescent health.

**Supplementary Information:**

The online version contains supplementary material available at 10.1186/s12889-024-19216-z.

## Introduction

Physical activity plays a crucial role in enhancing physical, mental, and cognitive health by protecting against metabolic, cardiovascular, and oncological diseases [1]. Conversely, a sedentary lifestyle is associated with adverse health effects including metabolic and musculoskeletal disorders, cancer, depression, and an increased risk of cardiovascular and all-cause mortality [2]. This association is attributed to metabolic, hemodynamic, and neurohormonal pathways [2]. Importantly, physical activity in children and adolescents yields diverse health benefits, with positive effects extending into adulthood [3–7]. Acknowledging this, the World Health Organization recommends that children and adolescents engage in at least 60 min of moderate-to-vigorous physical activity (MVPA) daily, including a minimum of three days of vigorous-intensity aerobic and muscle-strengthening activities. This recommendation also emphasizes the importance of reducing sedentary time with the overarching goal of optimizing health benefits [3].

However, according to data from the 2020 Korea Youth Risk Behavior Survey (KYRBS), only 5.9% of Korean adolescents met the recommended guidelines for aerobic exercise, and only 24% achieved muscle-strengthening exercise recommendations [8]. The situation is further alarming as there is a noticeable trend of increased sedentary time among adolescents, with approximately 45% and 37% reporting spending over 10 h sitting on weekdays and weekends, respectively [8]. A related study based on KYRBS data revealed that the prevalence of overweight and obesity among students aged 12 to 18 increased from 13.1% in 2005 to 23.4% in 2021. However, it was more pronounced in boys (15.0–29.0%) than in girls (10.9–17.3%) [9] and may be associated with reduced healthy physical activity and increased sedentary behavior.

The National Health Plan 2030 (HP2030) is a comprehensive policy framework established by the Ministry of Health and Welfare to present policy directions for promoting public health. It focuses on the following three goals to improve physical activity levels: (1) expansion and enhancement of local community resources and establishment of governance for physical activity, (2) development and provision of services to encourage physical activity for each target population, and (3) creation of a physical activity-friendly environment and strengthening of accessibility [10]. Objective monitoring of physical activity levels is essential to assess the efficacy of interventions targeted at enhancing physical activity levels in adolescents, which calls for physical activity surveillance to provide the basis for national planning for public health [11]. Moreover, because physical activity is a complex and multidimensional behavior, it is essential to gain a comprehensive understanding of the theoretical factors that influence physical activity levels [12, 13]. Given literature suggests that multifaceted factors, especially from a social-ecological model perspective, ranging from individual (e.g., demographic characteristics, knowledge, and attitudes) to community and political dimensions (e.g., community environment or services and national laws and policies), are intricately linked to physical activity levels [14, 15]. Therefore, the development of evaluative instruments is crucial for facilitating the assessment of these characteristics. These instruments can assess the effectiveness of interventions and identify key factors that influence physical activity, providing a better understanding of physical activity among adolescents and baseline information for health promotion plans.

Since 2005, the KYRBS has been an annual initiative overseen by the Korea Disease Control and Prevention Agency (KDCA) and the Ministry of Education, to assess adolescents’ health-related behaviors (e.g., tobacco and alcohol use, physical activity, dietary behaviors, use of a seat belt in the car, and safety education at school) related to non-communicable diseases and unintentional injuries [16]. This representative cross-sectional survey targets all middle and high school students (ages 12–18) in South Korea. The KYRBS is organized into three versions: mandatory, rotating, and optional. Mandatory questions must be answered annually, whereas rotating questions recur every three years. Optional questions, rather than periodic surveys, are introduced when timely demand increases. Additionally, in-depth surveys for specific areas are also conducted on a rotating basis to reduce the burden on respondents. This structure was designed to meet the growing demand for diverse health indicators while maintaining efficiency. The mandatory KYRBS questions focus on behaviors that should be monitored annually. These include instances of vigorous-intensity physical activity sustained for a minimum of 20 min, aerobic activities lasting at least 60 min that elevate heart rate, muscle-strengthening activities, and sedentary behavior. Conversely, the rotating questions evaluate behaviors that do not require yearly monitoring but require more comprehensive research. These rotating items include inquiries about walking habits (specifically, the number of days participants walked for at least 10 consecutive minutes), daily walking duration, frequency of physical education classes in the previous week, and the number of sports teams in which students regularly participated in the preceding semester. However, these items are limited in their ability to assess the factors that affect physical activity and to consider the intensity of physical activity to determine the overall level of physical activity in adolescents.

Therefore, this study developed and validated a series of in-depth rotating KYRBS questionnaires, explicitly designed to achieve a more profound understanding of the patterns and determinants of physical activity during adolescence. The questionnaire development process covered key areas such as diet, weight management, and physical activity. While a brief overview of the overarching development process for health behaviors, including diet, weight management, and physical activity, was reported in the KDCA’s Public Health Weekly Report [17], this study provides a more detailed exploration of the methodology employed, specifically in the development of a questionnaire focused on physical activity. Additionally, it presents findings on the current state of adolescent physical activity as revealed through the validity evaluation of the questionnaire.

## Methods

### Developing a questionnaire focused on physical activity

**Fig. 1:**
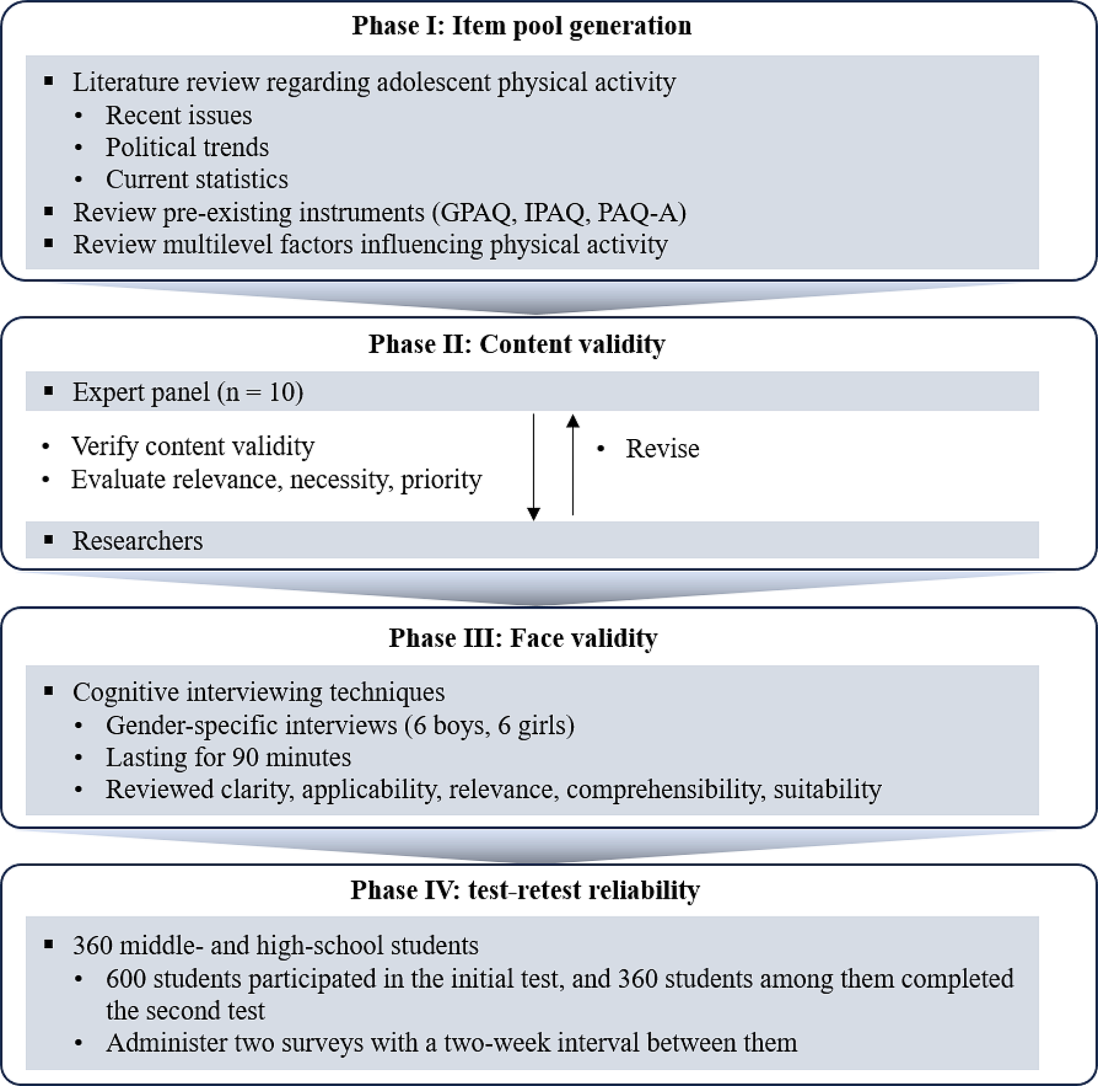
Development process flowchart for the questionnaire. GPAQ, General Physical Activity Questionnaire; IPAQ, International Physical Activity Questionnaire; PAQ-A, Physical Activity Questionnaire for Adolescents

The in-depth KYRBS questionnaires, aimed at assessing trends and determinants of physical activity among South Korean adolescents, underwent a comprehensive four-stage development and validation process: (1) creation of item pools, (2) expert verification of content validity, (3) face validity evaluation by adolescents, and (4) assessment of test-retest reliability for the finalized questionnaires (Fig. 1).

### Phase 1: item pool generation

In the initial stage, we initiated the development of an item pool through an extensive brainstorming session among the researchers, composed of adolescent health experts, preventive medicine specialists, and experts in the KYRBS. To identify relevant themes for assessment, we conducted a literature review, specifically focusing on recent issues related to adolescent physical activity mentioned on the internet and news, policy directions outlined in HP2030 [10] for promoting physical activity, and trends in physical activity among South Korean youth using data from the KYRBS from 2005 to 2020 [8, 9, 18]. Additionally, we performed a comprehensive review of preexisting domestic and international instruments, including the General Physical Activity Questionnaire (GPAQ) [19], International Physical Activity Questionnaire (IPAQ) [20, 21], and Physical Activity Questionnaire for Adolescents (PAQ-A) [22], to examine which specific items are contained within and to evaluate their applicability for integration. We conducted a thorough review of the multilevel factors influencing physical activity, drawing insights from a socio-ecological model [14, 23]. Ideally, interventions would target all dimensions of the socio-ecological model, including individual, household, parental, school, peer, community, sociocultural, and political levels, to effectively enhance physical activity. However, given resource constraints, it is crucial to identify the most influential levels within specific contexts to develop the most effective strategies. To this end, we tried to develop questions aimed at identifying key factors, thereby aiding in the development of targeted and resource-efficient strategies to enhance physical activity.

### Phase 2: content validity

Content validity is related to how well the questions in a questionnaire represent the full theoretical concept being assessed [24]. The content validity of the initial draft of the questionnaire was assessed by a panel of experts comprising 10 members. This panel comprised preventive medicine specialists, family medicine practitioners, and behavioral health professionals responsible for evaluating the questionnaire items in terms of their relevance, necessity, and priority. Additionally, they were asked to provide feedback to further refine the questionnaire. A revised version of the questionnaire was developed based on this input. The revised questionnaire was reevaluated by an expert panel and further refined by the researchers as necessary. This iterative process ensured that the revised questionnaire had robust content validity.

### Phase 3: face validity

Face validity refers to the subjective assessment of whether a questionnaire or instrument appears appropriate and contains relevant items at first glance [25]. This evaluation helps determine if a question seems suitable for measuring the intended concept. Face validity was evaluated using cognitive interview techniques [26], an evidence-based, qualitative method designed to determine whether the draft survey questions, covering attitudes, behaviors, or factual information, were interpreted as intended by the targeted population. Assuming lower comprehension levels in younger individuals, we specifically targeted 13-year-olds, the youngest adolescent group. We selected the sample from a national frame maintained by a professional research company, using a stratified random sampling approach to ensure representation across different regions and sexes. Given the coronavirus disease 2019 (COVID-19) situation, virtual rather than in-person interviews were carried out. Written informed consent was obtained from both the students and their parents. These sessions, facilitated by a trained moderator, probed the cognitive processing of participants when responding to the survey questions. Two sex-specific cognitive interviews were conducted to ensure homogeneous interview groups, each lasting 90 min, with participants providing consent for audio recordings to comprehensively capture the discussions. Prior to the interviews, participants independently completed the paper-based survey and personally recorded the time taken to respond to each question. During the interview sessions, participants were prompted to share feedback on the clarity, applicability, relevance, comprehensibility, and suitability of the questions. They were asked questions such as “Were there any words or sentences that were difficult to understand?” and “Did you encounter any difficulties while responding?” Based on the responses, the moderator facilitated discussions with unstructured questions to explore a variety of opinions. Participants were also encouraged to offer insights and recommendations regarding individual survey items. Following these feedback sessions, revisions were made as necessary to all items, particularly focusing on the question sequence, language use, and wording of individual items. By listening again to the audio recordings, we analyzed each response to pinpoint specific difficulties encountered by participants, such as issues with the wording of the questions, the context, or the response options, which informed targeted revisions aimed at constructing clearer and more effective questions. Through this iterative process conducted separately within male and female groups, the survey was able to achieve face validity by aligning with the understandings and perspectives of the target population.

### Phase 4: test-retest reliability

The items adjusted following the face validity test were used to conduct a test-retest reliability analysis. Test-retest reliability refers to the capability of a scale to yield consistent scores across different time points within a population that remains stable [27]. Random participant selection was implemented using a national youth sample frame owned by a professional survey company, with stratification by grade, sex, and region to ensure a nationally representative sample. We aimed to recruit 50 participants from each sex (male, female) and grade level (from 1st year of middle school to 3rd year of high school), resulting in a total of 600 participants for the initial test. This sample size accommodated the anticipated dropout rates after the initial test. Utilizing the KYRBS’s online platform, participants engaged in self-administered online surveys. The first survey was conducted from May 19 to June 1, 2022; followed by the second, approximately two weeks later, from June 2 to June 13, 2022. The students completed the survey at home and did so independently, without supervision. Test-retest reliability was determined using the intraclass correlation coefficient (ICC) for continuous variables and kappa coefficients for categorical variables. Interpreting the results, values from 0.01 to 0.2 signify slight agreement, 0.21 to 0.40 denote fair agreement, 0.41 to 0.60 express moderate agreement, 0.61 to 0.80 communicate substantial agreement, and 0.81 to 1.0 indicate almost perfect agreement [28, 29]. The coefficient of internal consistency for multi-item scales was assessed using Cronbach’s alpha [30]. Results yielding a Cronbach’s alpha value of over 0.7 were interpreted as indicative of high internal consistency [31]. It was determined that items with an ICC of 0.21 or above would be included in the questionnaire based on the opinion of researchers and a panel of experts. However, exceptions were made for items deemed essential by experts, such as those related to factors facilitating or inhibiting physical activity, which were included even if their ICC fell below the set threshold. All analyses were performed using R 4.1.2 (R Foundation for Statistical Computing, Vienna, Austria).

### Evaluating the current state of adolescent physical activity in Korea

To assess the current physical activity levels of Korean adolescents, we analyzed the responses of 600 adolescents who participated in the initial evaluation of test-retest reliability. For categorical variables, we calculated the frequency and percentage, while for continuous variables, we computed the mean (SD) and median (Q1-Q3).

### Ethical considerations

The study protocol was approved by the Institutional Review Board of Ewha Womans University Seoul Hospital (SEUMC 2022-03-029-002). Written informed consent was obtained from all participants before any study-related procedures were initiated.

## Results

### Item pool generation

Based on the comprehensive review that incorporated recent topics relevant to adolescent health, longitudinal trends in health behaviors, and insights from previously validated questionnaires, 20 preliminary items across four key themes were developed.

Analysis of historical data from the KYRBS [8, 9, 18, 32] during the process of survey development revealed low levels of physical activity and a downward trend in the proportion of individuals engaging in high-intensity physical activity for more than three days per week (37.8% in 2018, 32% in 2019, and 27.5% in 2020). The 2020 KYRBS data showed that only 14% of individuals participated in physical activity for at least 60 min/day for more than five days per week. The proportion of adolescents engaged in aerobic and muscle-strengthening activities that met or exceeded the World Health Organization 2020 exercise guidelines for adolescents did not exceed 30%. A sex-based disparity was also evident, with 19.9% of boys meeting the criteria compared with 7.7% of girls. To enhance physical activity levels, accurate measurement of physical activity is essential. Therefore, items measuring both the number of days and the duration of vigorous- and moderate-intensity physical activity were designed in alignment with established protocols from validated questionnaires such as the GPAQ and IPAQ. This method facilitates the calculation of metabolic equivalents of tasks (METs), offers an objective measure, and helps in the promotion of adequate physical activity among youths.

The need for revitalizing school sports clubs was recognized as a crucial objective for HP2030 to enhance physical activity among youths [10]. A questionnaire was thus developed to capture the occurrence and duration of physical activity in schools, covering both physical education classes and school sports clubs. Additionally, new survey items were added to assess physical activity outside the school setting, including after school hours and on weekends, to provide a more comprehensive understanding of the factors influencing physical activity levels among adolescents.

Items related to the frequency of walking or cycling to and from school, along with the associated challenges, and the availability of community physical activity infrastructure were developed in alignment with HP2030’s goal of creating a “health-friendly environment,” specifically focusing on the “expansion and enhancement of local community resources and establishment of governance” [10].

Furthermore, the factors that either inhibit or promote physical activity, as well as the level of parental encouragement, were explored to identify moderators of physical activity. These items were developed based on the social-ecological model [14, 23], enabling us to identify factors that hinder or facilitate physical activity across multiple levels, such as individual, household and parental, school and peer, community, and sociocultural-political factors. These comprehensive reviews and suggestions resulted in the development of 20 preliminary items across four key themes, designed to understand the broader context of physical activity among youths.

### Content validity

The initial draft of the questionnaire underwent two rounds of expert consultations for content validity assessment, resulting in various modifications: eight items were discarded, three were added, and twelve were modified following expert recommendations [Supplementary Table 1]. This process led to the development of an in-depth questionnaire comprising 15 items distributed across five themes: level of physical activity (7 items), school sports club activities (2 items), transportation-related physical activity (2 items), physical activity-promoting environments (1 item), and physical activity-mediating factors (3 items).

### Face validity

To evaluate face validity, a group of 12 participants comprising 6 males and 6 females, aged 13 years old, were selected. We confirmed whether the questions were phrased in terms understandable to adolescents and checked whether, regardless of their place of residence or sex, the questions were understood by the adolescents as intended. Feedback, queries, comprehension, and comments from participants led to improvements in the clarity, sequencing, and wording of the 15 items. To enhance the understanding of terms such as “high-intensity exercise” and “moderate-intensity exercise,” examples of high-intensity and moderate-intensity exercises were provided. Additionally, to clarify questions about physical activity frequency, a method for counting physical activity frequency was added. Furthermore, to ensure that everyone understands and responds to the term “free time” in the same way, the phrase “excluding school physical education time and intra-school sports team-related exercise time” was added in parentheses next to the term “free time.” It was indicated that there were no issues with understanding the other aspects. The face validity of the final revision was confirmed with 12 adolescents.

### Test-retest reliability

The initial survey involved 600 respondents, 300 each from middle and high schools, spanning grades one to three, with ages ranging from 13 to 19 years old. Of them, 360 completed the second survey, resulting in a response rate of approximately 60%. Table 1 provides the participants’ demographics. The retest subsample exhibited no significant differences in age, sex, or school level compared with the original participants. The ICCs of the two-week retest reliability of the items ranged from 0.29 to 0.70. Among the 15 items, three demonstrated substantial internal consistency (frequency of vigorous- and moderate-intensity physical activities and encouragement from parents to engage in physical activity), six showed moderate consistency (duration of vigorous- and moderate-intensity physical activities, duration of exercising or playing sports during an average physical education class, frequency of in-school physical activity outside of physical education class for the past seven days, frequency of physical activity after school or on weekends for the past seven days, duration of exercising or playing sports during school sports club activities among students who are participating in school sports club, and reasons for not participating in a sports team among students who are not participating in school sports club), and six exhibited fair consistency (the remaining items). Findings from the test-retest reliability assessment have been reported in a prior study [17].

**Table 1:** Demographic characteristics of participants who completed the test-retest reliability evaluation. Numbers are presented as n (%)

		First survey (*n* = 600)	Second survey (*n* = 360)	*p*
Sex				1.000
	Male	300 (50.0)	180 (50.0)	
	Female	300 (50.0)	180 (50.0)	
Age				0.999
	13 years	102 (17.0)	62 (17.2)	
	14 years	99 (16.5)	60 (16.7)	
	15 years	97 (16.2)	56 (15.6)	
	16 years	105 (17.5)	62 (17.2)	
	17 years	103 (17.2)	63 (17.5)	
	18 years	91 (15.2)	56 (15.6)	
	19 years	3 (0.5)	1 (0.3)	
School level				1.000
	Middle school	300 (50.0)	180 (50.0)	
	High school	300 (50.0)	180 (50.0)	

We developed two distinct sets of questions to evaluate the frequency and duration of vigorous- and moderate-intensity physical activity lasting 10 min or more in the past week (Q1-1 and Q2-1 of Supplementary Material). This information enables the calculation of the METs, an objective measure that allows for comparisons of physical activity levels. Question Type 1 required participants to specify the exact number of minutes spent on vigorous or moderate physical activity on each day of the past week. Contrastingly, Question Type 2 required participants to indicate the average number of minutes they exercised per day on occasions when they engaged in vigorous or moderate physical activity for over 10 min at a time in the last seven days. The METs calculated for both question types are presented in Table 2. The findings demonstrated that Question Type 2 generally exhibited higher test-retest reliability than Question Type 1. Cronbach’s alpha values for both vigorous- and moderate-intensity physical activity suggested a high or relatively high degree of internal consistency across three parameters: exercise frequency (0.80 and 0.75), exercise duration (0.64 and 0.66), and METs (0.67 and 0.68). Each question type had its own set of advantages and disadvantages. Inquiring about the amount of time spent exercising over the past seven days by day of the week (Question Type 2) is believed to generate more accurate responses, as it does not require respondents to calculate their average weekly exercise time themselves, unlike Question Type 1, which might lead to approximate estimations. However, although it may yield more precise data, it could be more time-consuming because respondents must record their daily exercise amounts individually, potentially leading to lower response rates due to the detailed tracking required. Additionally, the opinion of the expert panel on both types of questions was unbiased; as such, both were presented as viable options. The final in-depth questionnaire is included in the Supplementary Material.

**Table 2:** Vigorous or moderate physical activity in the past seven days

	Exercise frequency (days)		Exercise duration (minutes)		Metabolic equivalents of task	
	Type 1	Type 2	Type 1	Type 2	Type 1	Type 2
**Vigorous-intensity physical activity**						
Median (Q1–Q3)	3 (2–4)	3 (1–5)	40 (10–60)	40 (20–60)	680 (140–1,440)	960 (320–1,920)
Test-retest reliability	0.70	0.65	0.50	0.69	0.56	0.70
Cronbach’s alpha	0.80		0.65		0.67	
**Moderate-intensity physical activity**						
Median (Q1–Q3)	3 (2–5)	3 (1–6)	30 (20–60)	32 (16–52)	720 (240–1,440)	880 (320–1,680)
Test-retest reliability	0.61	0.61	0.46	0.61	0.51	0.79
Cronbach’s alpha	0.75		0.66		0.68	

Type 1: Questions regarding the number of days and average daily duration of vigorous or moderate activity exceeding 10 min within the past week

Type 2: Questions regarding the duration of vigorous or moderate physical activity on each individual day over the past week

### The current state of adolescent physical activity in Korea

The physical activity patterns of South Korean adolescents were analyzed using responses from a sample of 600 students who participated in the initial survey for test-retest reliability assessment. As reported in Tables 3, 7.2% of participants engaged in vigorous-intensity physical activity for at least five days per week. Conversely, 19.8% of students did not engage in such activities, with an additional 16.6% participating only once a week. The mean duration of daily vigorous-intensity physical activity was approximately 60 min (median: 50 min). For moderate-intensity physical activity, 21.5% of students participated for at least five days per week. Contrastingly, 15.9% of participants did not participate in these activities, whereas 15.2% engaged in them only once a week. The average daily duration of moderate-intensity activities was approximately 50 min (median: 40 min).

**Table 3:** Physical activity patterns of 600 students

Items		*n* (%) or mean (SD)/median (Q1-Q3)
**Theme 1. Levels of physical activity**		
**Frequency (days) of vigorous-intensity physical activities**		
	Never	119 (19.8)
	Once per week	100 (16.6)
	Twice per week	114 (19.0)
	Three days per week	115 (19.2)
	Four days per week	49 (8.2)
	Five days per week	51 (8.5)
	Six days per week	7 (1.2)
	Everyday	45 (7.5)
**Duration (minutes) of vigorous-intensity physical activities**		59.6 (57.7) / 50 (30–60)
**Frequency (days) of moderate-intensity physical activities**		
	Never	95 (15.9)
	Once per week	91 (15.2)
	Twice per week	125 (20.8)
	Three days per week	101 (16.8)
	Four days per week	59 (9.8)
	Five days per week	65 (10.8)
	Six days per week	13 (2.2)
	Everyday	51 (8.5)
**Duration (minutes) of moderate-intensity physical activities**		53.4 (57.4) / 40 (30–60)
***Theme 2. School sports club activities***		
**Duration (minutes) of exercising or playing sports during an average physical education class**		
	No physical education class	13 (2.2)
	Less than 10 min	26 (4.3)
	10 min to 19 min	51 (8.5)
	20 min to 29 min	97 (16.2)
	More than 30 min	413 (68.8)
**Frequency of in-school physical activity outside of physical education class for the past 7 days**		
	Never	249 (41.5)
	Once per week	78 (13.0)
	Twice per week	83 (13.8)
	Three days per week	73 (12.2)
	Four days per week	30 (5.0)
	Five days per week	49 (8.2)
	Six days per week	3 (0.5)
	Everyday	35 (5.8)
**Frequency (days) of physical activity after school or on weekends for the past 7 days**		
	Never	234 (39.0)
	Once per week	105 (17.5)
	Twice per week	83 (13.8)
	Three days per week	62 (10.4)
	Four days per week	32 (5.3)
	Five days per week	39 (6.5)
	Six days per week	12 (2.0)
	Everyday	33 (5.5)
**Duration (minutes) of exercising or playing sports during school sports club activities among students who are participating in school sports club** (***n* = 334)**		
	Not physically active	20 (6.0)
	Less than 10 min	15 (4.5)
	10 min to 19 min	31 (9.3)
	20 min to 29 min	50 (15.0)
	More than 30 min	218 (65.2)
**Reasons for not participating in a sports club among students who are not participating in school sports club** (***n* = 266)**		
	Dislike of exercise	70 (26.3)
	To do something else during that time	77 (28.9)
	No friends to participate in together	15 (5.7)
	No team to join, or sports teams are not activated in school	82 (30.8)
	The participation (registration) fee	1 (0.4)
	Participation in sports outside of school	2 (0.8)
	Others	19 (7.1)
***Theme 3. Transportation-related physical activity***		
**Frequency of walking or bicycling for the past 7 days**		
	Never	87 (14.5)
	Once per week	23 (3.8)
	Twice per week	33 (5.5)
	Three days per week	49 (8.2)
	Four days per week	24 (4.0)
	Five days per week	94 (15.7)
	Six days per week	42 (7.0)
	Everyday	248 (41.3)
**Reason for not walking or biking to get to and from places**		
	Walk or ride a bike always	127 (21.3)
	Long distance to move	181 (30.2)
	A lot of stuff to carry	79 (13.2)
	To move faster	90 (15.0)
	No pedestrian or bicycle paths	33 (5.5)
	Physical disability (e.g., leg injury)	21 (3.5)
	Others	69 (11.5)
***Theme 4. Physical activity-promoting environments***		
**Physical activity related infrastructure in community**		
	Yes	556 (92.7)
	No	44 (7.3)
***Theme 5. Factors mediating physical activity***		
**Factors encouraging physical activity (1st place)**		
	To stay healthy	171 (28.5)
	To have fun	195 (32.5)
	To relieve stress	33 (5.5)
	To build muscle	25 (4.2)
	To lose weight	80 (13.3)
	Recommended by a teacher	15 (2.5)
	Recommended by parents or siblings	18 (3.0)
	To hang out with friends	44 (7.3)
	To get into college or to improve grades	11 (1.8)
	Others	8 (1.4)
**Factors hindering physical activity (1st place)**		
	Physical disability	44 (7.3)
	No interest in exercise	131 (21.8)
	Prefer to do other things during leisure time	142 (23.7)
	To focus on studies	138 (23.0)
	Participation fee	12 (2.0)
	No friends to exercise with	50 (8.3)
	No sports facility nearby	22 (3.7)
	Owing to COVID-19	51 (8.5)
	Others	10 (1.7)
**Encourage physical activity by parents or guardians**		
	Very often	45 (7.5)
	Often	149 (24.8)
	Sometimes	232 (38.7)
	Rarely	174 (29.0)

Concerning school-based physical activities, 6.5% of students exercised for less than 10 min during physical education classes and 10.5% in school sports clubs. Notably, 44.3% of students did not participate in school sports clubs, citing reasons such as the absence of active sports clubs within the school (30.8%), prioritizing academics or other activities (28.9%), and a dislike of exercise (26.3%). Additionally, 41.5% of students reported non-engagement in leisure-time physical activities during school hours, and 39% of students did not participate in physical activity or exercise outside of school hours during both weekdays and weekends.

Approximately 64% of students reported commuting via walking or bicycling to various destinations at least five days per week, while 14.5% abstained from such activities. Among those who did not walk or cycle, the primary reasons included lengthy distances (30.2%), the necessity of quicker modes of transport (15.0%), and perceived inconvenience or difficulty (13.2%). Notably, most students (92.7%) reported having access to proximate exercise facilities.

The primary factors motivating physical activity among students were personal enjoyment (32.5%), health benefits (28.5%), and weight management goals (13.3%). However, the main barriers to physical activity were a preference for different leisure activities (23.7%), academic obligations (23.0%), and aversion to exercise (21.8%). Regarding parental or guardian influence, 32.3% of students reported frequent encouragement from their parents or guardians to participate in physical activity, while 67.7% stated that such encouragement was received sometimes or rarely.

## Discussion

This study aimed to achieve two main objectives: first, to develop an in-depth survey questionnaire focused on adolescent physical activity; and second, to identify adolescents’ physical activity levels by applying the developed in-depth questionnaire. To this end, we generated themes and in-depth items for the KYRBS, guided by a thorough review of recent issues, existing statistics, political agendas, and international survey instruments concerning adolescent physical activity. After rigorous evaluation of content validity, face validity, and test-retest reliability of the initially proposed items, revisions were made based on the feedback received. Finally, we generated 15 items that were categorized into five distinct themes: (1) Levels of physical activity, (2) School sports club activities, (3) Transportation-related physical activity, (4) Physical activity-promoting environments, and (5) Factors mediating physical activity. This developed survey is intended to provide valuable evidence for researchers, health professionals, and policymakers to identify the status of, and trends in, adolescents’ physical activity, as well as to facilitate the assessment of interventions, strategies, and programs aimed at enhancing physical activity among adolescents.

MVPA is a crucial indicator of physical health, necessitating comprehensive analysis that includes both the frequency of exercise and the average daily duration over a seven-day period for high- and moderate-intensity activities. Although the KYRBS currently assesses the frequency of high-intensity physical activity days per week, it does not include questions about the duration of high-intensity physical activity or the frequency and duration of moderate-intensity physical activities. This lack of detail in the survey makes it challenging to calculate METs, which are essential for quantifying total energy expenditure associated with physical activities. To bridge this gap and enhance the accuracy of physical activity assessment, we developed items designed to capture both the number of days and the duration spent on high- and moderate-intensity physical activities in a week. Implementing these measures will facilitate more precise evaluations of physical activity levels and related health outcomes among Korean youth. Our approach is aligned with the goals of HP2030 [10], which aims to increase the rate of aerobic physical activity levels in Korean youth. These enhanced survey items are fundamental to our primary research objectives of creating a robust tool for evaluating physical activity levels in this population.

The 2023 School Sports Promotion Plan in Korea [33] highlights the importance of diversifying operational models and programs for school sports clubs and promoting a student-led sports culture. To support this initiative, there is a growing emphasis on not only increasing the frequency but also the intensity of physical education classes and participation in school sports clubs to boost physical activity among adolescents [34, 35]. The KYRBS currently tracks how frequently students attend physical education classes and participate in school sports clubs on a weekly basis. However, this data alone does not provide a full picture of the impact of these activities on student health. To address this gap, our study has introduced new survey items that measure the average duration (in minutes) of activity during these sessions. This additional metric is essential for not only assessing the frequency but also the intensity of physical activity, thereby offering a more comprehensive understanding of how these activities contribute to overall physical activity levels among students. Furthermore, by separately analyzing the duration and frequency of both physical education classes and school sports club participation, we can obtain detailed insights into the specific roles each type of activity plays in promoting physical health. This granular analysis is pivotal for developing more targeted and effective health interventions. Such data is crucial for supporting the objectives of Korea’s School Sports Promotion Plan, which aims to diversify and enhance the effectiveness of school sports programs. This ultimately helps in developing initiatives that are more likely to increase the effectiveness of school-based physical activities, fostering a healthier and more active youth population in Korea [36, 37].

To align with the HP2030 objectives that emphasize fostering active lifestyles among children and adolescents and enhancing school sports clubs, we introduced new survey questions to understand why students do not participate in school sports clubs. According to the preliminary findings from the survey conducted on 600 students for test-retest reliability assessment, 30.8% of students do not participate in school sports clubs primarily because their schools lack such clubs, most likely due to inadequate funding [33]. Allocating more budget is expected to expand the number of school sports clubs, thereby improving access to sports activities and reducing the prevalence of schools without active sports clubs.

In this study, we sought to develop survey items to deepen our understanding of the factors influencing physical activity among Korean youth. To achieve this, we employed the social-ecological model [14, 23], which provides a comprehensive framework for analyzing the multilayered influences on behaviors. This model helped us investigate the various levels at which physical activity can be promoted. Based on this model, our survey items were specifically designed to explore barriers and facilitators across five domains: individual, household and parental, school and peer, community, and sociocultural-political factors. One particular focus was the role of parents or guardians as mediators in promoting physical activity. Preliminary findings from a survey of 600 students, conducted to test the reliability of the survey, revealed that 29% of parents or guardians seldom encouraged their children to participate in physical activities. This highlights a significant need to enhance parental awareness and involvement. Additionally, we introduced new items to explore reasons for non-participation in school sports, alternative transportation methods to walking or biking, and other factors that may impede physical activity. These questions are tailored to clearly identify what promotes or hinders physical activity within the specific context in which the survey is applied. By pinpointing specific barriers and facilitators at each level, and reflecting on the contextual factors, our study establishes a solid foundation for designing targeted, theory-based interventions. These interventions aim to address the complexities affecting adolescent physical activity and promote sustainable behavior changes in line with the HP2030 objectives. This approach marks a significant advancement over international questionnaires such as the GPAQ [19], IPAQ [20, 21], and PAQ-A [22], providing a more nuanced understanding of physical activity determinants in specific cultural and environmental contexts.

We surveyed a representative sample of 600 adolescents using an in-depth questionnaire developed for this study. Only 8% of the surveyed students met the World Health Organization’s recommended guidelines [33] for engaging in a minimum of 60 min of moderate or vigorous exercise daily. The majority of students (nearly 70%) cited various reasons for their lack of physical activity, including a preference for alternative activities, academic obligations, and a general dislike of exercise. Additionally, approximately 40% of students reported no engagement in physical activity during their free time, whether during school, after school, or on weekends. These findings underscore the limitations of strategies that rely solely on students’ independent decisions to exercise, highlighting obstacles to increasing overall physical activity levels. The data emphasize the importance of enhancing the quality and effectiveness of physical education classes and school sports clubs to facilitate daily participation in at least 60 min of moderate-intensity physical activity, regardless of personal preferences. The survey further revealed that 6.5% and 10.5% of students reported engaging in less than 10 min of physical activity during school physical education classes and sports clubs, respectively. Additionally, over 30% indicated the inactivity of school sports clubs as a primary deterrent to participation. Therefore, it is crucial to enforce regulations that ensure an adequate duration of physical activity and mandate the activation of school sports clubs. Incorporating enjoyable components into these programs can provide additional motivation for students and foster a physical activity culture [38].

This study has several strengths. First, a robust and reliable questionnaire was developed by a thorough examination of face and content validity as well as test-retest reliability. We ensured face validity by testing the instrument on adolescents as young as 13 years, a group that is more likely to have lower literacy skills than older adolescents. In addition, for the test-retest reliability evaluation, a representative sample was selected based on grade, sex, and geographical location. Consequently, we generated items that were comprehensible to most adolescents. It has been shown that our survey demonstrates validity and reliability that are not inferior to other international surveys in methodological terms. Modified PAQ-A survey results showed acceptable internal consistency and test-retest reliability, but weak-to-moderate correlations with objectively assessed moderate-to-vigorous physical activity, self-reported fitness, and self-efficacy [39]. A systematic review of various physical activity questionnaires for adolescents revealed that the qualitative measurement of the questionnaires lacked acceptable levels of reliability and validity [40, 41]. In addition, the previous study using the Korean version of IPAQ among adolescents and adults aged 15 to 69 reported reliabilities of 0.381 for vigorous-intensity and 0.531 for moderate-intensity physical activity days [42], while our study showed higher reliabilities (kappa = 0.70 for vigorous-intensity, 0.61 for moderate-intensity) [17]. Second, we developed items that can discern complex patterns related to adolescent physical activity, taking into account multilevel influencing factors. Identifying the levels at which factors either inhibit or promote adolescent physical activity within the socio-ecological model, and targeting those specific levels for intervention, could lead to more cost-effective strategies for improving physical activity among adolescents.

Nonetheless, this study has some limitations. First, exploratory factor analysis was not conducted because a single item was developed per theme. This analysis would have allowed researchers to assess the internal consistency among the components within a factor [43]. Second, because it is a self-report questionnaire, it is prone to reporting errors and social desirability bias [44]. It is important to note that self-reported surveys do not accurately measure actual physical activity levels, which may affect the accuracy of reported prevalence rates. Third, while there are potential sex differences in physical activity levels among adolescents [45, 46], our analysis reported the prevalence for the total population, which could overlook these sex-specific variations.

Regardless of the limitations, this study contributes significantly to the understanding of adolescent physical activity patterns and offers valuable insights for developing targeted interventions to promote physical activity and improve overall health among Korean adolescents. Investigating the current prevalence of physical activity among adolescents provides crucial insights into their health behaviors, informs policy-making, and helps in designing targeted interventions to improve physical activity levels and overall health. Therefore, the 15 items developed in this study can function as effective tools to investigate the patterns and potential mediators of adolescent physical activity. Moreover, utilizing a survey based on this questionnaire can offer valuable evidence for formulating intervention programs aimed at elevating awareness, establishing environments conducive to physical activity, and enhancing levels of physical activity among adolescents.

## Conclusions

Drawing from a life course approach [47], adequate physical activity during adolescence could enhance adult health outcomes and reduce disease prevalence. This underscores the critical need to understand and evaluate current levels of physical activity among adolescents to inform the development of targeted early health intervention policies. In this study, we developed and validated in-depth questionnaires that elucidate the factors governing adolescents’ current physical activity status and related health behaviors. Using the questionnaires developed in this study, we found that only 8% of the adolescents met the WHO’s guidelines for daily physical activity, highlighting a significant gap. Furthermore, we found that policy and educational program improvements and social support are necessary to promote physical activity. We believe that the comprehensive questionnaire developed here not only demonstrates robust validity and reliability but also holds significant potential to shape future public health policies. By providing crucial data, this in-depth questionnaire will aid in creating environments that encourage increased physical activity among adolescents, ultimately contributing to their long-term health benefits. Furthermore, the information gathered from this survey is vital for assessing the effectiveness of existing policies and interventions, allowing them to be adapted based on actual needs and outcomes. Therefore, we believe that our research has practical implications for enhancing public health strategies aimed at improving the physical and overall health of future generations.

### Electronic supplementary material

Below is the link to the electronic supplementary material.

Supplementary Material 1

Supplementary Material 2

## Data Availability

The datasets used and/or analyzed in the current study are available from the corresponding author upon reasonable request.

## Abbreviations

MVPA: moderate-to-vigorous intensity physical activity
KYRBS: Korea Youth Risk Behavior Survey
KDCA: Korea Disease Control and Prevention Agency
HP2030: Health Plan 2030
GPAQ: General Physical Activity Questionnaire
IPAQ: International Physical Activity Questionnaire
ICC: intraclass correlation coefficient
METs: metabolic equivalents of task
PAQ-A: Physical Activity Questionnaire for Adolescents

## References

[1] 2018 Physical Activity Guidelines Advisory Committee. 2018 Physical Activity Guidelines Advisory Committee Scientific Report. U.S. Department of Health and Human Services. 2018. https://health.gov/sites/default/files/2019-09/PAG_Advisory_Committee_Report.pdf. Accessed 22 April 2024.

[2] Park JH, Moon JH, Kim HJ, Kong MH, Oh YH (2020). Sedentary lifestyle: overview of updated evidence of potential health risks. Korean J Fam Med.

[3] Bull FC, Al-Ansari SS, Biddle S, Borodulin K, Buman MP, Cardon G (2020). World Health Organization 2020 guidelines on physical activity and sedentary behaviour. Br J Sports Med.

[4] Tremblay MS, Carson V, Chaput JP, Connor Gorber S, Dinh T, Duggan M (2016). Canadian 24-hour movement guidelines for children and youth: an integration of physical activity, sedentary behaviour, and sleep. Appl Physiol Nutr Metab.

[5] Telama R, Yang X, Viikari J, Välimäki I, Wanne O, Raitakari O (2005). Physical activity from childhood to adulthood: a 21-year tracking study. Am J Prev Med.

[6] Sawyer SM, Afifi RA, Bearinger LH, Blakemore S-J, Dick B, Ezeh AC (2012). Adolescence: a foundation for future health. Lancet.

[7] Sato M, Kodama S, Sugawara A, Saito K, Sone H (2009). Physical fitness during adolescence and adult mortality. Epidemiology.

[8] Korea Centers for Disease Control and Prevention. Korean Youth Health Risk Behavior Web-Based Survey. Korea Centers for Disease Control and Prevention. 2020. https://www.kdca.go.kr/yhs/. Accessed 1 May 2024.

[9] Ban C, Shin H, Eum S, Yon H, Lee S, Choi Y (2023). 17-year trends of body mass index, overweight, and obesity among adolescents from 2005 to 2021, including the COVID-19 pandemic: a Korean national representative study. Eur Rev Med Pharmacol Sci.

[10] Ministry of Health and Welfare, Korea Health Promotion Institute. Health Plan (Health Plan 2030). Ministry of Health and Welfare and Korea Health Promotion Institute. 2022. https://www.khepi.or.kr/healthplan. Accessed 31 March 2022. (in Korean).

[11] National Academies of Sciences, Engineering, and, Health M, and Medicine Division; Food and Nutrition Board; Committee on Strategies for Implementing Physical Activity Surveillance. Implementing strategies to enhance public health surveillance of physical activity in the United States. National Academies Press (US). May 21, 2019. https://www.ncbi.nlm.nih.gov/books/NBK545630/. Accessed 31 March 2022. 31454187

[12] King AC, Stokols D, Talen E, Brassington GS, Killingsworth R (2002). Theoretical approaches to the promotion of physical activity: forging a transdisciplinary paradigm. Am J Prev Med.

[13] Craggs C, Corder K, van Sluijs EM, Griffin SJ (2011). Determinants of change in physical activity in children and adolescents: a systematic review. Am J Prev Med.

[14] Hu D, Zhou S, Crowley-McHattan ZJ, Liu Z (2021). Factors that influence participation in physical activity in school-aged children and adolescents: a systematic review from the social ecological model perspective. Int J Environ Res Public Health.

[15] King KM, Gonzalez GB (2018). Increasing physical activity using an ecological model. ACSMs Health Fit J.

[16] Kim Y, Choi S, Chun C, Park S, Khang YH, Oh K (2016). Data resource profile: the Korea Youth Risk Behavior web-based Survey (KYRBS). Int J Epidemiol.

[17] Park HJ, Lee HA, Park B, Shin YH, Jun SH, Kim EJ (2022). Development of in-depth questionnaire items related to dietary behaviors, physical activity, obesity, and weight control efforts for the Korea Youth Risk Behavior Survey. Public Health Wkly Rep.

[18] Statistics Korea. Statistical Description Data Inquiry. Statistics Korea. https://www.k-stat.go.kr/metasvc/msba100/statsdcdta?statsConfmNo=117058&kosisYn=Y. Accessed 31 March 2022. (in Korean).

[19] de Courten M (2002). Developing a simple global physical activity questionnaire for population studies. Aust Epidemiol.

[20] Craig CL, Marshall AL, Sjöström M, Bauman AE, Booth ML, Ainsworth BE (2003). International physical activity questionnaire: 12-country reliability and validity. Med Sci Sports Exerc.

[21] Lee PH, Macfarlane DJ, Lam TH, Stewart SM (2011). Validity of the International Physical Activity Questionnaire Short Form (IPAQ-SF): a systematic review. Int J Behav Nutr Phys Act.

[22] Kowalski KC, Crocker PRE, Donen RM (2004). The physical activity questionnaire for older children (PAQ-C) and adolescents (PAQ-A) manual.

[23] Bronfenbrenner U (1977). Toward an experimental ecology of human development. Am Psychol.

[24] Schultz KS, Whitney DJ (2005). Measurement theory in action: case studies and exercises.

[25] Setia MS (2017). Methodology series module 9: designing questionnaires and clinical record forms - part II. Indian J Dermatol.

[26] Willis GB, Artino AR (2013). What do our respondents think we’re asking? Using cognitive interviewing to improve medical education surveys. J Grad Med Educ.

[27] Aaronson N, Alonso J, Burnam A, Lohr KN, Patrick DL, Perrin E, Stein RE (2002). Assessing health status and quality-of-life instruments: attributes and review criteria. Qual Life Res.

[28] McHugh ML (2012). Interrater reliability: the kappa statistic. Biochem Med.

[29] Landis JR, Koch GG (1977). The measurement of observer agreement for categorical data. Biometrics.

[30] Cronbach LJ (1951). Coefficient alpha and the internal structure of tests. Psychometrika.

[31] Erdim L, Ergün A, Kuğuoğlu S (2019). Reliability and validity of the Turkish version of the physical activity questionnaire for older children (PAQ-C). Turk J Med Sci.

[32] Kwon R, Koo MJ, Lee SW, Choi YS, Shin YH, Shin JU (2023). National trends in physical activity among adolescents in South Korea before and during the COVID-19 pandemic, 2009–2021. J Med Virol.

[33] Ministry of Education. Press release: MOE to promote school sports to reinforce healthy minds and bodies of all students. April 28. 2023. https://www.moe.go.kr/boardCnts/viewRenew.do?boardID=294&boardSeq=94620&lev=0&searchType=null&statusYN=W&page=1&s=moe&m=020402&opType=N. Accessed 31 March 2022. (in Korean).

[34] Lee E-J, So W-Y, Youn H-S, Kim J. Effects of school-based physical activity programs on health-related physical fitness of Korean adolescents: a preliminary study. Int J Environ Res Public Health. 202;18(6):2976. 10.3390/ijerph18062976. 10.3390/ijerph18062976 PMC7998220 33799424

[35] Barbry A, Carton A, Ovigneur H, Coquart J (2022). Relationships between sports club participation and health determinants in adolescents and young adults. Front Sports Act Living.

[36] van der Ploeg HP, Merom D, Chau JY, Bittman M, Trost SG, Bauman AE (2010). Advances in population surveillance for physical activity and sedentary behavior: reliability and validity of time use surveys. Am J Epidemiol.

[37] Sylvia LG, Bernstein EE, Hubbard JL, Keating L, Anderson EJ (2014). Practical guide to measuring physical activity. J Acad Nutr Diet.

[38] Rodríguez Macías M, Abad Robles MT, Giménez Fuentes-Guerra FJ (2021). Effects of sport teaching on students’ enjoyment and fun: a systematic review and meta-analysis. Front Psychol.

[39] Aggio D, Fairclough S, Knowles Z, Graves L (2016). Validity and reliability of a modified English version of the physical activity questionnaire for adolescents. Arch Public Health.

[40] Chinapaw MJM, Mokkink LB, van Poppel MNM, van Mechelen W, Terwee CB (2010). Physical activity questionnaires for youth: a systematic review of measurement properties. Sports Med.

[41] Hidding LM, Chinapaw MJM, van Poppel MNM, Mokkink LB, Altenburg TM (2018). An updated systematic review of childhood physical activity questionnaires. Sports Med.

[42] Oh JH, Yang YJ, Kim BS, Kang JH (2007). Validity and reliability of Korean version of International Physical Activity Questionnaire (IPAQ) short form. J Korean Acad Fam Med.

[43] Dabbagh A, Seens H, Fraser J, MacDermid JC (2023). Construct validity and internal consistency of the Home and Family Work roles questionnaires: a cross-sectional study with exploratory factor analysis. BMC Womens Health.

[44] Sallis JF, Saelens BE (2000). Assessment of physical activity by self-report: status, limitations, and future directions. Res Q Exerc Sport.

[45] Kretschmer L, Salali GD, Andersen LB, Hallal PC, Northstone K, Sardinha LB (2023). Sex differences in the distribution of children’s physical activity: evidence from nine countries. Int J Behav Nutr Phys Act.

[46] Steene-Johannessen J, Hansen BH, Dalene KE, Kolle E, Northstone K, Møller NC (2020). Variations in accelerometry measured physical activity and sedentary time across Europe-harmonized analyses of 47,497 children and adolescents. Int J Behav Nutr Phys Act.

[47] Wagner C, Carmeli C, Jackisch J, Kivimäki M, van der Linden BWA, Cullati S, Chiolero A (2024). Life course epidemiology and public health. Lancet Public Health.

