# Supplementary material for: Development and psychometric testing of a questionnaire for the Korea Youth risk behavior survey to assess physical activity behaviors
Source: BMC Public Health. 2024 Jun 24;24:1686. doi: 10.1186/s12889-024-19216-z (PMC11197320; doi:10.1186/s12889-024-19216-z)
Supplement: Supplementary file 1 — Supplementary Material 1 [file 12889_2024_19216_MOESM1_ESM.docx]

**Korea Youth Risk Behavior Survey: An in-depth questionnaire to assess physical activity**

**Q1. In the last seven days, how many days did you engage in vigorous-intensity physical activity for at least 10 minutes at a time, such that you were out of breath or sweating?**

※ Vigorous-intensity physical activities: jogging (running), soccer, basketball, singles tennis, taekwondo, hiking, cycling at a fast pace, swimming quickly, jumping rope, carrying heavy objects, aerobics, etc.

※ Include all physical activities throughout the day (physical education classes at school, sports club activities, physical education club activities, leisure-time physical activities, etc.).

1. None (→ Go to question 2)
2. 1 day per week (→ Go to question 1-1)
3. 2 days per week (→ Go to question 1-1)
4. 3 days per week (→ Go to question 1-1)
5. 4 days per week (→ Go to question 1-1)
6. 5 days per week (→ Go to question 1-1)
7. 6 days per week (→ Go to question 1-1)
8. 7 days per week (→ Go to question 1-1)

[For respondents who answered 2–8 for Question 1 on vigorous-intensity physical activity frequency]

▶ Option 1

**Q1-1. In the last seven days, if you have engaged in vigorous-intensity physical activity for at least 10 minutes at a time, such that you were out of breath or sweating, on average, how long did you spend per day on such activities?**

※ Vigorous intensity physical activities: jogging (running), soccer, basketball, singles tennis, taekwondo, hiking, cycling at a fast pace, swimming quickly, jumping rope, carrying heavy objects, aerobics, etc.

※ Example: If in the last seven days you engaged in vigorous intensity physical activity for more than 10 minutes on three days, and on those days you exercised for 10 minutes, 30 minutes, and 50 minutes respectively, you would calculate (10+30+50)/3 = 30 minutes.

| Per day: __ hours __ min |
| --- |

▶ Option 2

**Q1-1. In the last seven days, if you have engaged in vigorous-intensity physical activity for at least 10 minutes at a time, such that you were out of breath or sweating, on average, how long did you spend each day on such activities?**

※ Vigorous intensity physical activities: jogging (running), soccer, basketball, singles tennis, taekwondo, hiking, cycling at a fast pace, swimming quickly, jumping rope, carrying heavy objects, aerobics, etc.

※ Include all physical activities throughout the day (physical education classes at school, sports club activities, physical education club activities, leisure time physical activities, etc.).

| Day of the Week | Time |
| --- | --- |
| Mon | __ hours __ min |
| Tue | __ hours __ min |
| Wed | __ hours __ min |
| Thu | __ hours __ min |
| Fri | __ hours __ min |
| Sat | __ hours __ min |
| Sun | __ hours __ min |

**Q2. In the last seven days, how many days did you engage in moderate-intensity physical activity for at least 10 minutes at a time, such that you were slightly out of breath?**

※ Moderate-intensity physical activities: non-competitive volleyball, table tennis, doubles tennis, badminton, swimming at a slow pace, cycling at a slightly fast pace, brisk walking, carrying light objects, dancing (social dancing, contemporary dance, etc.).

※ Include all physical activities throughout the day (physical education classes at school, sports club activities, physical education club activities, leisure time physical activities, etc.).

1 None (→ Go to question 3)

2 1 day per week (→ Go to question 2-1)

3 2 days per week (→ Go to question 2-1)

4 3 days per week (→ Go to question 2-1)

5 4 days per week (→ Go to question 2-1)

6 5 days per week (→ Go to question 2-1)

7 6 days per week (→ Go to question 2-1)

8 7 days per week (→ Go to question 2-1)

[For respondents who answered 2–8 for Question 2 on moderate-intensity physical activity frequency]

▶ Option 1

**Q2-1. In the last seven days, if you have engaged in moderate-intensity physical activity for at least 10 minutes at a time, such that you were slightly out of breath, on average, how long did you spend per day on such activities?**

※ Moderate-intensity physical activities: non-competitive volleyball, table tennis, doubles tennis, badminton, swimming at a slow pace, cycling at a slightly fast pace, brisk walking, carrying light objects, dancing (social dancing, contemporary dance, etc.).

※ Example: If in the last seven days you engaged in moderate-intensity physical activity for more than 10 minutes on three days, and on those days you exercised for 10 minutes, 30 minutes, and 50 minutes respectively, you would calculate (10+30+50)/3 = 30 minutes.

| Per day: __ hours __ min |
| --- |

▶ Option 2

**Q2-1. In the last seven days, if you have engaged in moderate-intensity physical activity for at least 10 minutes at a time, such that you were slightly out of breath, how much time per day did you spend on such activities on those days?**

※ Moderate-intensity physical activities: non-competitive volleyball, table tennis, doubles tennis, badminton, swimming at a slow pace, cycling at a slightly fast pace, brisk walking, carrying light objects, dancing (social dancing, contemporary dance, etc.).

※ Include all physical activities throughout the day (physical education classes at school, sports club activities, physical education club activities, leisure time physical activities, etc.).

| Day of the Week | Time |
| --- | --- |
| Mon | __ hours __ min |
| Tue | __ hours __ min |
| Wed | __ hours __ min |
| Thu | __ hours __ min |
| Fri | __ hours __ min |
| Sat | __ hours __ min |
| Sun | __ hours __ min |

**Q3. On average, how many minutes per class do you spend exercising in the playground or gym during physical education classes at school?**

※ Exclude time spent on self-study or theoretical lessons inside the classroom.

1 No physical education classes

2 Less than 10 minutes

3 10 to less than 20 minutes

4 20 to less than 30 minutes

5 30 minutes or more

**Q4. In the last seven days, how many days did you engage in exercise or physical activities outside of physical education class at school (such as during breaks or lunchtime)?**

※ Exclude time spent on self-study or theoretical lessons inside the classroom.

1. None
2. 1 day per week
3. 2 days per week
4. 3 days per week
5. 4 days per week
6. 5 days per week
7. 6 days per week
8. 7 days per week

**Q5. In the last seven days, how many days did you engage in exercise or physical activities during weekends or after school hours?**

1. None
2. 1 day per week
3. 2 days per week
4. 3 days per week
5. 4 days per week
6. 5 days per week
7. 6 days per week
8. 7 days per week

[Branch question of a mandatory question on the number of sports club participation in this semester: for respondents who answered “0” for the mandatory question]

**Q6. What is the main reason you do not regularly participate in a school sports club?**

1. Because I dislike exercise
2. Because I prefer to study or do other things during that time
3. Because there is no one to do it with
4. Because there is no sports activity team at school that I want to join
5. Because of the participation (registration) fee
6. Because I am affiliated with a sports team outside of school or attend a physical education academy
7. Other

[Branch question of a mandatory question on the number of sports club participation in this semester: for respondents who selected “more than 1” for the mandatory question]

**Q7. On average, how much time per day do you spend actively exercising as part of a school sports club?**

※ Exclude time spent on self-study or theoretical lessons inside the classroom.

1. No active exercise involvement
2. Less than 10 minutes
3. 10 to less than 20 minutes
4. 20 to less than 30 minutes
5. 30 minutes or more

**Q8. In the last seven days, how many days did you walk or use a bicycle for transportation?**

※ Include all instances of transportation, such as commuting to and from school.

1. None
2. 1 day per week
3. 2 days per week
4. 3 days per week
5. 4 days per week
6. 5 days per week
7. 6 days per week
8. 7 days per week

**Q9. If you chose to use a car, bus, subway, or other means of transportation instead of walking or cycling, what was the reason?**

※ Include all instances of transportation, such as commuting to and from school.

1. Always walked or used a bicycle for transportation
2. The distance was too far
3. Because I have a lot of baggage or am inconvenienced
4. To move faster
5. Lack of pedestrian paths or bike lanes (unsafe to walk or cycle)
6. Due to physical discomfort (e.g., leg injury)
7. Other

**Q10. Do you have parks, sports fields, or other physical exercise facilities within walking distance from your home?**

1. Yes
2. No

**Q11. How often do your parents (or guardians) usually encourage you to exercise or engage in physical activities?**

1. Very often
2. Often
3. Sometimes
4. Rarely

**Q12. If you do not engage in exercise or physical activities during your free time, what is the reason?**

※ Please respond with your top three reasons, ranking them from 1st to 3rd priority.

※ Free time physical activities include autonomous physical activities during school breaks, lunchtime, after school, weekends, etc. (including sports teams and physical education academies outside of school)

1. Due to health or physical reasons (injury, lack of fitness, underlying illness, etc.)
2. Dislike of exercise or find it bothersome
3. Prefer doing other things in my free time
4. To focus on academic studies
5. The cost required for exercising is burdensome
6. No one to exercise with
7. Lack of nearby or desirable exercise facilities
8. Due to COVID-19
9. Other

| Rank | Number (Choose from 1–9) or None | |
| --- | --- | --- |
| 1st Priority |  | ○ None |
| 2nd Priority |  | ○ None |
| 3rd Priority |  | ○ None |

**Q13. If you engage in exercise or physical activities during your free time, what is the reason?**

※ Please respond with your top three reasons, ranking them from 1st to 3rd priority.

※ Free time physical activities include autonomous physical activities during school breaks, lunchtime, after school, weekends, etc. (including sports teams and physical education academies outside of school)

1 For health reasons such as improving fitness

2 Because it’s enjoyable

3 To relieve stress

4 To build muscle

5 To lose weight

6 On the recommendation of school teachers

7 Encouraged by family members like parents or siblings

8 To socialize with peers

9 For college admissions or managing academic grades

10 Other

| Rank | Number (Choose from 1–10) or None | |
| --- | --- | --- |
| 1st Priority |  | ○ None |
| 2nd Priority |  | ○ None |
| 3rd Priority |  | ○ None |
