# Supplementary material for: Development and psychometric testing of a questionnaire for the Korea Youth risk behavior survey to assess physical activity behaviors
Source: BMC Public Health. 2024 Jun 24;24:1686. doi: 10.1186/s12889-024-19216-z (PMC11197320; doi:10.1186/s12889-024-19216-z)
Supplement: Supplementary file 2 — Supplementary Material 2 [file 12889_2024_19216_MOESM2_ESM.docx]

**Supplementary Table 1.** Comparison between initial draft and modified questionnaires after content validity assessment.

| **Before** |  | **After** |
| --- | --- | --- |
| **Theme 1. Levels of physical activity** | | |
| Frequency of vigorous-intensity physical activities | modify | Frequency of vigorous-intensity physical activities |
| Duration of vigorous-intensity physical activities | modify | Duration of vigorous-intensity physical activities |
| Frequency of moderate-intensity physical activities | modify | Frequency of moderate-intensity physical activities |
| Duration of moderate -intensity physical activities | modify | Duration of moderate -intensity physical activities |
| Sedentary lifestyle | discarded |  |
|  | added | Duration of exercising or playing sports during an average physical education class |
| Frequency of in-school physical activity outside of physical education class for the past 7 days | modify | Frequency of in-school physical activity outside of physical education class for the past 7 days |
| Duration of in-school physical activity outside of physical education class for the past 7 days | discarded |  |
|  | added | Frequency of physical activity after school or on weekends for the past 7 days |
| Factors encouraging physical activity | modify and move to theme 5 |  |
| Factors hindering physical activity | modify and move to theme 5 |  |
| **Theme 2. School sports club activities** | | |
| Duration of exercising or playing sports during school sports club | modify | Duration of exercising or playing sports during school sports club |
| Reasons for not participating in a sports team | modify | Reasons for not participating in a sports club |
| Number of community sports club participating | discarded |  |
| Duration of exercising or playing sports at community sports clubs | discarded |  |
| Reasons for not participating in a community sports club | discarded |  |
| Whether exercise or play sports in a community by oneself | discarded |  |
| Duration of exercising or playing sports in a community by oneself | discarded |  |
| **3. Transportation-related physical activity** | | |
| Frequency of walking or bicycling for the past 7 days | modify | Frequency of walking or bicycling for the past 7 days |
| Reason for not walking or biking to get to and from places | modify | Reason for not walking or biking to get to and from places |
| Frequency of using transportation such as buses, cars, or trains for the past 7 days | discarded |  |
| **4. Physical activity-promoting environments** | | |
| Physical activity related infrastructure in community | modify | Physical activity related infrastructure in community |
| **5. Factors mediating physical activity** | | |
|  | moved | Factors encouraging physical activity |
|  | moved | Factors hindering physical activity |
|  | added | Encouragement to engage in physical activity by parents |
